# 1-(But-2-enyl­idene)-2-(2-nitro­phen­yl)hydrazine

**DOI:** 10.1107/S1600536809037179

**Published:** 2009-09-19

**Authors:** Zhi-Gang Yin, Heng-Yu Qian, Xue-Wen Zhu, Chun-Xia Zhang

**Affiliations:** a Key Laboratory of Surface and Interface Science of Henan, School of Materials and Chemical Engineering, Zhengzhou University of Light Industry, Zhengzhou 450002, People’s Republic of China

## Abstract

The mol­ecule of the title Schiff base compound, C_10_H_11_N_3_O_2_, adopts an *E* geometry with respect to the C=N double bond. The mol­ecule is roughly planar, with the largest deviation from the mean plane being 0.111 (2) Å, The enyl­idene-hydrazine group is, however, slightly twisted with respect to the phenyl ring, making a dihedral angle of 6.5 (3)°. An intra­molecular N—H⋯O hydrogen bond may be responsible for the planar conformation. An inter­molecular N—H⋯O hydrogen bond links two mol­ecules around an inversion center, building a pseudo dimer.

## Related literature

For the role played by Schiff base compounds in the development of various proteins and enzymes, see: Kahwa *et al.* (1986 ▶); Santos *et al.* (2001 ▶).
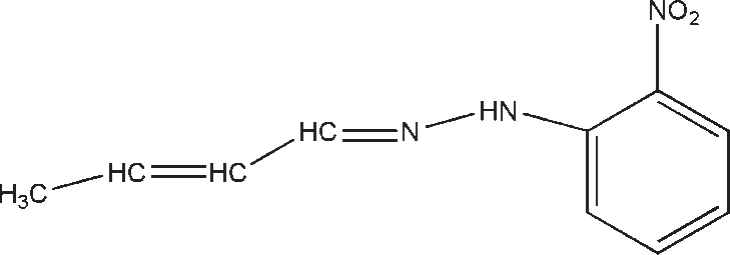


## Experimental

### 

#### Crystal data

C_10_H_11_N_3_O_2_
*M* *_r_* = 205.22
Triclinic, 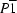

*a* = 4.2390 (6) Å
*b* = 11.456 (2) Å
*c* = 11.9840 (17) Å
α = 113.271 (15)°
β = 96.534 (12)°
γ = 95.595 (13)°
*V* = 524.64 (16) Å^3^
*Z* = 2
Mo *K*α radiation
μ = 0.09 mm^−1^
*T* = 296 K
0.25 × 0.19 × 0.18 mm

#### Data collection

Bruker SMART CCD area-detector diffractometer
Absorption correction: multi-scan (*SADABS*; Bruker, 1998 ▶) *T*_min_ = 0.979, *T*_max_ = 0.982
3321 measured reflections
1758 independent reflections
587 reflections with *I* > 2σ(*I*)
*R*_int_ = 0.039

#### Refinement

*R*[*F* ^2^ > 2σ(*F* ^2^)] = 0.039
*wR*(*F* ^2^) = 0.080
*S* = 0.68
1758 reflections
137 parameters
H-atom parameters constrained
Δρ_max_ = 0.12 e Å^−3^
Δρ_min_ = −0.11 e Å^−3^

### 

Data collection: *SMART* (Bruker, 1998 ▶); cell refinement: *SAINT* (Bruker, 1998 ▶); data reduction: *SAINT*; program(s) used to solve structure: *SHELXS97* (Sheldrick, 2008 ▶); program(s) used to refine structure: *SHELXL97* (Sheldrick, 2008 ▶); molecular graphics: *ORTEPIII* (Burnett & Johnson, 1996 ▶), *ORTEP-3 for Windows* (Farrugia, 1997 ▶) and *PLATON* (Spek, 2009 ▶); software used to prepare material for publication: *SHELXL97*.

## Supplementary Material

Crystal structure: contains datablocks global, I. DOI: 10.1107/S1600536809037179/dn2487sup1.cif

Structure factors: contains datablocks I. DOI: 10.1107/S1600536809037179/dn2487Isup2.hkl

Additional supplementary materials:  crystallographic information; 3D view; checkCIF report

## Figures and Tables

**Table 1:** Hydrogen-bond geometry (Å, °)

*D*—H⋯*A*	*D*—H	H⋯*A*	*D*⋯*A*	*D*—H⋯*A*
N2—H2*A*⋯O2	0.86	2.00	2.615 (3)	127
N2—H2*A*⋯O2^i^	0.86	2.53	3.353 (3)	160

Symmetry code: (i) 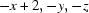.

**Fig. 1.**
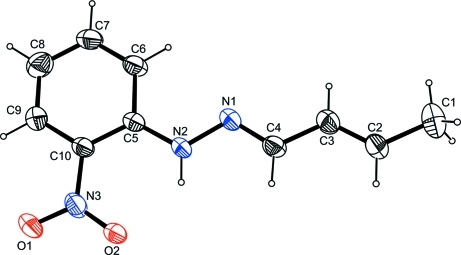
Molecular view of (I) with the atomlabeling scheme. Displacement ellipsoids are drawn at the 30% probability level. H atoms are represented as small spheres of arbitrary radii.

**Fig. 2.**
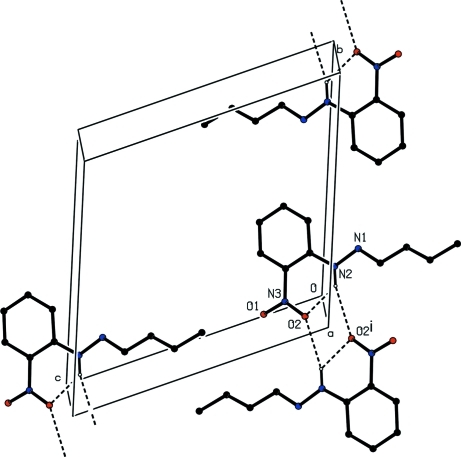
Partial packing of (I), showing the intra and intermolecular hydrogen bonds as dashed lines. H atoms not involved in hydrogen bonds have been omitted for clarity. [Symmetry codes: (i) -x+2, -y, -z]

## supplementary crystallographic information

### Comment

The chemistry of Schiff base has attracted a great deal of interest in recent years. These compounds play an important role in the development of various proteins and enzymes (Kahwa *et al.*, 1986; Santos *et al.*, 2001). As part of our in the study of the coordination chemistry of Schiff bases, we synthesized the title compound and determined its crystal structure.

The molecule is roughly planar with the largest deviation from the mean plane being -0.111 (2) Å at O1 (Fig. 1). The enylidene-hydrazine group is however slightly twisted with respect to the phenyl ring making a dihedral angle of 6.5 (3)° .

Intramolecular N—H···O bond may be responsible for the planar conformation whereas intermolecular N—H···O links two molecules around the inversion center buiding a pseudo dimer (Table 1, Fig. 2).

### Experimental

2-Nitrophenylhydrazine (1 mmol, 0.153 g) was dissolved in anhydrous ethanol (15 ml), The mixture was stirred for several minitutes at 351 K, but-2-enal (1 mmol, 0.070 g) in ethanol (8 mm l) was added dropwise and the mixture was stirred at refluxing temperature for 2 h. The product was isolated and recrystallized from methanol, red single crystals of (I) was obtained after 3 d.

### Refinement

H atoms were placed in calculated position and treated as riding with C—H = 0.93 Å(aromatic), 0.96 Å(methyl) and N—H = 0.86Å with *U*_iso_(H) = 1.2*U*_eq_(C,N) or *U*_iso_(H) = 1.5*U*_eq_(methyl).

### Crystal data

**Table:**

C_10_H_11_N_3_O_2_	*Z* = 2
*M**_r_* = 205.22	*F*(000) = 216
Triclinic, *P*1	*D*_x_ = 1.299 Mg m^−^^3^
Hall symbol: -P 1	Mo *K*α radiation, λ = 0.71073 Å
*a* = 4.2390 (6) Å	Cell parameters from 1958 reflections
*b* = 11.456 (2) Å	θ = 3.2–26.0°
*c* = 11.9840 (17) Å	µ = 0.09 mm^−^^1^
α = 113.271 (15)°	*T* = 296 K
β = 96.534 (12)°	Block, red
γ = 95.595 (13)°	0.25 × 0.19 × 0.18 mm
*V* = 524.64 (16) Å^3^	

### Data collection

**Table:**

Bruker SMART CCD area-detector diffractometer	1758 independent reflections
Radiation source: fine-focus sealed tube	587 reflections with *I* > 2σ(*I*)
graphite	*R*_int_ = 0.039
ω scans	θ_max_ = 25.0°, θ_min_ = 3.2°
Absorption correction: multi-scan (*SADABS*; Bruker, 1998)	*h* = −5→4
*T*_min_ = 0.979, *T*_max_ = 0.982	*k* = −13→12
3321 measured reflections	*l* = 0→14

### Refinement

**Table:**

Refinement on *F*^2^	Primary atom site location: structure-invariant direct methods
Least-squares matrix: full	Secondary atom site location: difference Fourier map
*R*[*F*^2^ > 2σ(*F*^2^)] = 0.039	Hydrogen site location: inferred from neighbouring sites
*wR*(*F*^2^) = 0.080	H-atom parameters constrained
*S* = 0.68	*w* = 1/[σ^2^(*F*_o_^2^) + (0.0315*P*)^2^] where *P* = (*F*_o_^2^ + 2*F*_c_^2^)/3
1758 reflections	(Δ/σ)_max_ < 0.001
137 parameters	Δρ_max_ = 0.12 e Å^−^^3^
0 restraints	Δρ_min_ = −0.11 e Å^−^^3^

### Special details

**Table:**

Geometry. All esds (except the esd in the dihedral angle between two l.s. planes) are estimated using the full covariance matrix. The cell esds are taken into account individually in the estimation of esds in distances, angles and torsion angles; correlations between esds in cell parameters are only used when they are defined by crystal symmetry. An approximate (isotropic) treatment of cell esds is used for estimating esds involving l.s. planes.
Refinement. Refinement of *F*^2^ against ALL reflections. The weighted *R*-factor *wR* and goodness of fit *S* are based on *F*^2^, conventional *R*-factors *R* are based on *F*, with *F* set to zero for negative *F*^2^. The threshold expression of *F*^2^ > σ(*F*^2^) is used only for calculating *R*-factors(gt) *etc*. and is not relevant to the choice of reflections for refinement. *R*-factors based on *F*^2^ are statistically about twice as large as those based on *F*, and *R*- factors based on ALL data will be even larger.

### Fractional atomic coordinates and isotropic or equivalent isotropic displacement parameters (Å^2^)

**Table:**

	*x*	*y*	*z*	*U*_iso_*/*U*_eq_	
C1	1.2639 (7)	0.1906 (3)	−0.4898 (3)	0.0923 (12)	
H1A	1.1868	0.2699	−0.4786	0.138*	
H1B	1.1535	0.1237	−0.5661	0.138*	
H1C	1.4905	0.2007	−0.4916	0.138*	
C2	1.2028 (7)	0.1556 (3)	−0.3856 (3)	0.0637 (10)	
H2	1.2765	0.0820	−0.3851	0.076*	
C3	1.0549 (7)	0.2191 (3)	−0.2947 (3)	0.0601 (10)	
H3	0.9757	0.2916	−0.2958	0.072*	
C4	1.0060 (7)	0.1857 (3)	−0.1944 (3)	0.0549 (9)	
H4	1.0791	0.1129	−0.1913	0.066*	
C5	0.6836 (7)	0.2802 (3)	0.0774 (2)	0.0451 (9)	
C6	0.6046 (6)	0.4007 (3)	0.0883 (3)	0.0584 (9)	
H6	0.6489	0.4308	0.0293	0.070*	
C7	0.4654 (7)	0.4732 (3)	0.1834 (3)	0.0681 (10)	
H7	0.4153	0.5520	0.1879	0.082*	
C8	0.3955 (8)	0.4328 (4)	0.2745 (3)	0.0767 (11)	
H8	0.3012	0.4840	0.3394	0.092*	
C9	0.4679 (7)	0.3169 (3)	0.2666 (3)	0.0641 (10)	
H9	0.4228	0.2883	0.3265	0.077*	
C10	0.6101 (7)	0.2408 (3)	0.1686 (2)	0.0497 (9)	
N1	0.8634 (6)	0.2541 (2)	−0.1088 (2)	0.0553 (7)	
N2	0.8205 (5)	0.2108 (2)	−0.01925 (19)	0.0548 (8)	
H2A	0.8807	0.1395	−0.0246	0.066*	
N3	0.6751 (6)	0.1190 (3)	0.1671 (2)	0.0634 (8)	
O1	0.5896 (6)	0.0865 (2)	0.2464 (2)	0.0933 (9)	
O2	0.8241 (5)	0.05249 (18)	0.08917 (18)	0.0722 (7)	

### Atomic displacement parameters (Å^2^)

**Table:**

	*U*^11^	*U*^22^	*U*^33^	*U*^12^	*U*^13^	*U*^23^
C1	0.096 (3)	0.119 (3)	0.074 (2)	0.006 (2)	0.027 (2)	0.051 (2)
C2	0.075 (3)	0.063 (2)	0.062 (2)	0.006 (2)	0.0186 (19)	0.0343 (19)
C3	0.069 (3)	0.065 (3)	0.057 (2)	0.014 (2)	0.011 (2)	0.0350 (19)
C4	0.063 (2)	0.052 (2)	0.057 (2)	0.0116 (19)	0.0080 (18)	0.0305 (19)
C5	0.048 (2)	0.041 (2)	0.049 (2)	0.0052 (18)	0.0036 (17)	0.0236 (18)
C6	0.064 (2)	0.053 (2)	0.072 (2)	0.014 (2)	0.0155 (17)	0.0372 (19)
C7	0.084 (3)	0.048 (3)	0.082 (2)	0.017 (2)	0.022 (2)	0.032 (2)
C8	0.080 (3)	0.071 (3)	0.082 (3)	0.025 (2)	0.028 (2)	0.026 (2)
C9	0.079 (3)	0.063 (3)	0.059 (2)	0.016 (2)	0.0244 (18)	0.030 (2)
C10	0.057 (2)	0.045 (2)	0.056 (2)	0.0113 (19)	0.0112 (17)	0.0282 (18)
N1	0.068 (2)	0.056 (2)	0.0565 (16)	0.0143 (15)	0.0179 (15)	0.0347 (16)
N2	0.076 (2)	0.052 (2)	0.0548 (16)	0.0199 (16)	0.0241 (15)	0.0353 (15)
N3	0.073 (2)	0.073 (2)	0.0618 (18)	0.0175 (18)	0.0187 (15)	0.0431 (18)
O1	0.149 (2)	0.084 (2)	0.0911 (17)	0.0467 (17)	0.0603 (16)	0.0652 (16)
O2	0.1081 (19)	0.0611 (18)	0.0802 (14)	0.0410 (15)	0.0499 (13)	0.0479 (13)

### Geometric parameters (Å, °)

**Table:**

C1—C2	1.495 (4)	C6—C7	1.356 (3)
C1—H1A	0.9600	C6—H6	0.9300
C1—H1B	0.9600	C7—C8	1.392 (4)
C1—H1C	0.9600	C7—H7	0.9300
C2—C3	1.314 (3)	C8—C9	1.361 (3)
C2—H2	0.9300	C8—H8	0.9300
C3—C4	1.429 (3)	C9—C10	1.399 (3)
C3—H3	0.9300	C9—H9	0.9300
C4—N1	1.276 (3)	C10—N3	1.442 (3)
C4—H4	0.9300	N1—N2	1.371 (3)
C5—N2	1.351 (3)	N2—H2A	0.8600
C5—C10	1.392 (3)	N3—O1	1.227 (3)
C5—C6	1.411 (3)	N3—O2	1.232 (3)
			
C2—C1—H1A	109.5	C5—C6—H6	119.4
C2—C1—H1B	109.5	C6—C7—C8	121.7 (3)
H1A—C1—H1B	109.5	C6—C7—H7	119.1
C2—C1—H1C	109.5	C8—C7—H7	119.1
H1A—C1—H1C	109.5	C9—C8—C7	118.7 (3)
H1B—C1—H1C	109.5	C9—C8—H8	120.7
C3—C2—C1	126.1 (3)	C7—C8—H8	120.7
C3—C2—H2	116.9	C8—C9—C10	120.1 (3)
C1—C2—H2	116.9	C8—C9—H9	119.9
C2—C3—C4	125.2 (3)	C10—C9—H9	119.9
C2—C3—H3	117.4	C5—C10—C9	121.9 (3)
C4—C3—H3	117.4	C5—C10—N3	121.8 (3)
N1—C4—C3	121.1 (3)	C9—C10—N3	116.3 (3)
N1—C4—H4	119.5	C4—N1—N2	116.1 (2)
C3—C4—H4	119.5	C5—N2—N1	119.9 (2)
N2—C5—C10	124.5 (3)	C5—N2—H2A	120.0
N2—C5—C6	119.1 (3)	N1—N2—H2A	120.0
C10—C5—C6	116.4 (3)	O1—N3—O2	121.7 (3)
C7—C6—C5	121.1 (3)	O1—N3—C10	118.9 (3)
C7—C6—H6	119.4	O2—N3—C10	119.4 (3)

### Hydrogen-bond geometry (Å, °)

**Table:**

*D*—H···*A*	*D*—H	H···*A*	*D*···*A*	*D*—H···*A*
N2—H2A···O2	0.86	2.00	2.615 (3)	127
N2—H2A···O2^i^	0.86	2.53	3.353 (3)	160

Symmetry codes: (i) −*x*+2, −*y*, −*z*.
